# Notfallversorgung – Reformbedarf aus ambulanter Sicht

**DOI:** 10.1007/s00108-022-01382-0

**Published:** 2022-07-27

**Authors:** Dominik von Stillfried, Sandra Mangiapane

**Affiliations:** Zentralinstitut für die kassenärztliche Versorgung in der Bundesrepublik Deutschland, Salzufer 8, 10587 Berlin, Deutschland

**Keywords:** Abrechnungsdaten, Überlastung von Notaufnahmen, Gesundheitsreform, Integrierte Notfallzentren, Medizinische Ersteinschätzung, Claims data, Emergency departments/crowding, Health care reform, Integrated emergency centers, Triage

## Abstract

Seit Jahren stehen Reformen der Akut- und Notfallversorgung auf der gesundheitspolitischen Agenda. So auch im aktuellen Koalitionsvertrag. Zur Einordnung der Ausgangslage werden ein inhaltlicher Rückblick sowie eine Analyse der Abrechnungsdaten aus der ambulanten und stationären Notfallversorgung vorgenommen. Folgt man der Hypothese, dass bisherige Reformansätze insbesondere auf einen Anstieg ambulant vertragsärztlich behandelbarer Fälle in der Notfallversorgung reagierten, muss die Fallzahlentwicklung in der Dekade bis zum Pandemiebeginn (2010–2019) zur Kenntnis genommen werden. Sie zeigt eine Stagnation der Fallzahl in Notaufnahmen und einen seit 2016 rückläufigen Trend insbesondere bei ambulanten Notfallbehandlungen durch Krankenhäuser. Dieser Effekt kann nicht zweifelsfrei auf bereits eingeführte Maßnahmen zur Patientensteuerung (Bereitschaftspraxen, Telefontriage) zurückgeführt werden. Die Analyse der Abrechnungsdiagnosen spricht dafür, dass eine klare Arbeitsteilung zwischen Bereitschaftspraxen und Notaufnahmen besteht. Die konsistente Verlagerung der Fälle hochbetagter Patienten in die ambulante Versorgung durch Notaufnahmen legt aber eine notwendige Weiterentwicklung dieser Arbeitsteilung nahe. Weitere Maßnahmen der Steuerung durch Weiterleitung von Patienten aus Notaufnahmen in die vertragsärztliche Versorgung müssen noch im Detail beschlossen und umgesetzt werden (§ 120 Abs. 3b Sozialgesetzbuch [SGB] V). In der Diskussion wird die Auffassung vertreten, dass Implementierung und Evaluation dieser Maßnahmen abgewartet werden sollten, bevor weitergehende Reformschritte ergriffen werden.

## Abstract (en)

Reforms of emergency and after hours care have been on the health policy agenda for years, and continue to remain there in the present government’s program. In order to outline where this originates from, we provide a brief summary of past reform steps and a claims data analysis of ambulatory and inpatient emergency and after hours care. Given that previous reform steps have been triggered by increases in emergency department utilization by low acuity cases that could have been treated by office-based physicians during office hours or in after hours care, we analyze the ambulatory and inpatient claims data in the past decade prior to the pandemic (2010–2019). The result shows increases until 2015. Thereafter, the overall case load in emergency wards has levelled, while outpatient cases in emergency wards have decreased. This effect cannot safely be attributed to elements of demand management such as after hours practices in hospitals and telephone triage that have been implemented since then. The distribution of diagnostic codes in claims data suggests that emergency wards and after hours practices have reached a clear division of labor. A continuous shift of patients in age groups 70+ into ambulatory care in emergency wards suggests that this division of labor needs to be further elaborated. Past legislation has not yet been fully implemented. In particular, it is necessary to decide which triage system emergency wards will need to use to identify low acuity patients for redirection into ambulatory care. We argue that implementation and evaluation of these requirements should be completed before further far reaching reforms are enacted.

Der aktuelle Koalitionsvertrag greift Kerngedanken, namentlich die integrierten Notfallzentren (INZ), aus einem in der letzten Legislaturperiode nicht weiter verfolgten Gesetzentwurf zur Notfallreform wieder auf. Im vorliegenden Beitrag wird untersucht, wie sich der Reformbedarf zwischenzeitlich entwickelt hat. Werden noch bestehende gesetzliche Arbeitsaufträge zur Verbesserung der Patientensteuerung umgesetzt, könnte das Ziel einer Entlastung der Notaufnahmen der Krankenhäuser um vertragsärztlich behandelbare Patienten bereits weitgehend erreicht werden.

## Einordnung bisheriger Reformansätze in der Notfallversorgung

An der Notfallversorgung sind das Rettungswesen, die Notaufnahmen der Krankenhäuser und die Vertragsärzte durch den ärztlichen Bereitschaftsdienst (ÄBD) zu den sprechstundenfreien Zeiten beteiligt. Die Sicherstellung der flächendeckenden vertragsärztlichen Versorgung auch zu den sprechstundenfreien Zeiten – das Sozialgesetzbuch (SGB) V spricht hier von Notdienst – liegt in der Zuständigkeit der Kassenärztlichen Vereinigungen (KVen). Dazu sollen die KVen neben der Organisation des ÄBD auch mit zugelassenen Krankenhäusern kooperieren. Dies umfasst die Einrichtung von Notdienstpraxen in oder an Krankenhäusern sowie die unmittelbare Einbindung der Notfallaufnahmen der Krankenhäuser in den Notdienst. Darüber hinaus sind gesetzlich Versicherte in Deutschland gemäß § 76 Abs. 1 Satz 2 SGB V im Notfall berechtigt, selbstständig eine Notaufnahme aufzusuchen – auch während der Sprechstundenzeiten. Soweit in diesem Fall eine ambulante Behandlung ausreicht, zählt auch diese zum Notdienst. Mit dem einschränkenden Charakter von § 76 Abs. 1 Satz 2 SGB V, der nur im Notfall zur selbstständigen Inanspruchnahme berechtigt, geht die Einschränkung einher, dass die ambulante Notfallbehandlung nur Maßnahmen umfasst, die erforderlich sind, bis eine Behandlung im Rahmen der Regelversorgung erfolgen kann.

Eine deutlich steigende Direktinanspruchnahme der Krankenhausnotaufnahmen wird daher in mehrfacher Hinsicht als Indikator für Reformbedarf gesehen. Aus Sicht der Krankenhäuser werden die Ressourcen der Notfallversorgung nicht kostendeckend beansprucht und oftmals überlastet. In der wissenschaftlichen Literatur wird die Überfüllung von Notaufnahmen (Crowding) als Ursache für Qualitätseinbußen der Notfallversorgung beschrieben. Ziel gesetzlicher Interventionen ist daher die Entlastung der Notfallversorgung um vertragsärztlich behandelbare Patienten. Die zuletzt in Kraft getretenen Gesetze setzen dabei sowohl am Sicherstellungsauftrag der KVen als auch an Pflichten der Krankenhäuser an und haben das gemeinsame Ziel, Notfälle präziser zu definieren und Akutfälle aus der Notfallversorgung in den Bereitschaftsdienst oder in die vertragsärztliche Regelversorgung zu steuern.

Einen wesentlichen Diskussionsbeitrag zum Reformbedarf der Notfallversorgung leistete eine 2015 veröffentlichte Studie der Deutschen Gesellschaft Interdisziplinäre Notfall- und Akutmedizin [1]. In einer retrospektiven Analyse kamen die Autoren unter anderem zu dem Ergebnis, dass rund 30 %, bei entsprechender Ressourcenverfügbarkeit sogar bis zu etwa 50 % der selbsteinweisenden Patienten vertragsärztlich behandelbar gewesen wären. Patientenbefragungen ergeben im Wesentlichen drei Arten von Inanspruchnahmegründen: mangelndes Wissen über Alternativen, tatsächlich oder vermeintlich eingeschränkte Verfügbarkeit der vertragsärztlichen Versorgung, Annahmen über eine höhere Behandlungsqualität oder Geschwindigkeit in Notaufnahmen [2, 3]. Auch in der internationalen Literatur wurde ein Anstieg selbsteinweisender Patienten in Notaufnahmen festgestellt, wobei als Instrumente einer verbesserten Patientensteuerung insbesondere Bereitschaftspraxen an Krankenhäusern, eine niedrigschwellige Telefontriage bzw. die Kombination beider Maßnahmen vorgeschlagen wurden [4].

Der Gesetzgeber hat den Sicherstellungsauftrag der Kassenärztlichen Vereinigungen mehrfach erweitert

Um das Problem der Fehlinanspruchnahme von Notaufnahmen zu adressieren, hat der Gesetzgeber den Sicherstellungsauftrag der KVen mehrfach erweitert. Mit dem Krankenhausstrukturgesetz (KHSG; 2015) wurden die KVen verpflichtet, Portalpraxen an Notaufnahmen einzurichten; zur Sicherstellung des Bereitschaftsdiensts sollen die KVen mit Krankenhäusern kooperieren. Mit dem Terminservice- und Versorgungsgesetz (TSVG; 2019) wurden KVen verpflichtet, unter der Rufnummer 116117 rund um die Uhr allen Anrufern mit akuten Beschwerden eine angemessene medizinische Versorgung zu vermitteln, die auch eine telemedizinische Behandlung umfassen kann. Die Vermittlung des Versorgungsangebots soll auf Grundlage eines einheitlichen und strukturierten telefonischen Ersteinschätzungsverfahrens erfolgen. Die KVen sollen mit den Rettungsleitstellen kooperieren.

Ein im Januar 2020 vorgelegter Gesetzentwurf zur Reform der Notfallversorgung [5] bezog sich auf Vorschläge des Sachverständigenrats von 2018 [6] und zielte auf eine Neuordnung der Zusammenarbeit zwischen Rettungsdienst, Krankenhäusern und vertragsärztlicher Versorgung. Dazu sollte eine gesetzliche Unterscheidung zwischen Notfall und Akutfall eingeführt werden und die Unterscheidung im konkreten Einzelfall nach Maßgabe strukturierter Ersteinschätzungsverfahren erfolgen. So sollten KVen auf Antrag von Rettungsleitstellen mit diesen verbindlich gemeinsame Notfallleitsysteme bilden, um die vertragsärztliche Versorgung von Akutfällen sicherzustellen, die das Rettungswesen gemäß Ersteinschätzung unberechtigt in Anspruch nehmen. Mit Krankenhäusern sollten KVen INZ bilden, um rund um die Uhr eine vertragsärztliche Versorgung von Akutfällen sicherzustellen, die gemäß Ersteinschätzung keiner Notfallbehandlung bedürfen. Nach Verbändeanhörung im Februar 2020 wurde das Gesamtvorhaben einer umfassenden Notfallreform dann allerdings zurückgestellt, sodass der vorläufig letzte Reformschritt in der mit dem Gesundheitsversorgungsweiterentwicklungsgesetz (GVWG) eingeführten Neuregelung von § 120 Abs. 3b SGB V liegt [7]. Dabei wurden Krankenhäuser verpflichtet, bei selbsteinweisenden Patienten künftig vor Aufnahme einer Behandlung mittels eines strukturierten Ersteinschätzungsverfahrens zu prüfen, ob eine vertragsärztliche Behandelbarkeit vorliegt. Der Gemeinsame Bundesausschuss (G-BA) soll hierzu bis Jahresmitte 2022 Vorgaben machen. Danach sollen ambulante Notfallbehandlungen in Krankenhäusern nur noch vergütet werden, wenn gemäß Vorgaben des G‑BA zur Durchführung der Ersteinschätzung ein sofortiger Behandlungsbedarf festgestellt wurde. Der Nachweis gilt als Voraussetzung für die Abrechnung einer ambulanten Behandlung in der Notaufnahme. Zudem soll der G‑BA auch Vorgaben zur Weiterleitung geeigneter Patienten in die vertragsärztliche Versorgung machen.

Somit wurde der Schwerpunkt auf eine bessere Fallsteuerung zwischen Notfall- und Akutversorgung mittels Ersteinschätzungsverfahren gelegt. Auf die Einrichtung von INZ als einem neuen Versorgungsstrukturelement wurde dagegen ebenso verzichtet wie auf Maßnahmen zur Entlastung des Rettungsdiensts. Der aktuelle Koalitionsvertrag unterstellt weiterhin Reformbedarf und greift namentlich insbesondere die Einführung von INZ als Zielvorstellung einer Reform auf.

## Bisherige Entwicklung der Inanspruchnahme

Da der Bedarf für weitergehende Reformen meist mit der zunehmenden Fehlinanspruchnahme der Notfallversorgung begründet wird, ist ein Blick auf die jüngere Entwicklung der Inanspruchnahme notwendig. Zur Abschätzung der Fallzahlentwicklung in der ambulanten Notfallversorgung wurden die Abrechnungsdaten der KVen für den Bereitschaftsdienst ausgewertet, zur Entwicklung der stationären Notfallversorgung die DRG-Statistik (*DRG* „diagnosis-related group“), in der ungeplante Aufnahmen ausgewiesen sind. Da die Inanspruchnahme der Notaufnahmen während der Pandemie starken Sondereffekten unterlag, werden die Jahre 2020 und 2021 hier ausgeklammert [9].

### Entwicklung der Fallzahlen

In der Dekade bis zum Beginn der Pandemie (2010–2019) stagnierte der Fallzahlanstieg in den Notaufnahmen bereits ab 2016 (Tab. 1). Dabei waren ab 2016 insbesondere die ambulanten Notfälle in den Krankenhäusern rückläufig (Tab. 2), während sie im ÄBD anstiegen. Insgesamt nahmen die ambulanten Notfälle bis 2018 zu. Fokussiert man auf die Differenzierung nach Sprechstundenzeit (Tab. 3), waren die ambulanten Notfälle in Notaufnahmen während der Sprechstundenzeit bereits ab 2015 rückläufig, außerhalb der Sprechstundenzeiten ab 2016.

**Table 1:** 

Jahr	Anzahl Fälle		
	Ambulant (EBM)	Stationär (DRG)	Gesamt
2010	8.491.745 (100 %)	6.844.022 (100 %)	15.335.767 (100 %)
2011	8.821.536 (104 %)	7.163.214 (105 %)	15.984.750 (104 %)
2012	8.972.319 (106 %)	7.464.171 (109 %)	16.436.490 (107 %)
2013	9.919.838 (117 %)	7.798.904 (114 %)	17.718.742 (116 %)
2014	10.268.242 (121 %)	8.107.676 (118 %)	18.375.918 (120 %)
2015	10.372.858 (122 %)	8.395.822 (123 %)	18.768.680 (122 %)
2016	10.673.947 (126 %)	8.608.710 (126 %)	19.282.657 (126 %)
2017	10.518.857 (124 %)	8.649.277 (126 %)	19.168.134 (125 %)
2018	10.413.834 (123 %)	8.650.121 (126 %)	19.063.955 (124 %)
2019	10.272.213 (121 %)	8.745.168 (128 %)	19.017.381 (124 %)

*DRG* „diagnosis-related group“, *EBM* Einheitlicher Bewertungsmaßstab

**Table 2:** 

Jahr	Anzahl Behandlungsfälle nach EBM		
	Notaufnahmen	ÄBD	Gesamt
2010	8.491.745 (100 %)	8.869.272 (100 %)	17.361.017 (100 %)
2011	8.821.536 (104 %)	8.770.335 (99 %)	17.591.871 (101 %)
2012	8.972.319 (106 %)	8.741.350 (99 %)	17.713.669 (102 %)
2013	9.919.838 (117 %)	9.440.038 (106 %)	19.359.876 (112 %)
2014	10.268.242 (121 %)	8.916.275 (101 %)	19.184.517 (111 %)
2015	10.372.858 (122 %)	8.667.283 (98 %)	19.040.141 (110 %)
2016	10.673.947 (126 %)	8.762.790 (99 %)	19.436.737 (112 %)
2017	10.518.857 (124 %)	8.770.523 (99 %)	19.289.380 (111 %)
2018	10.413.834 (123 %)	9.053.522 (102 %)	19.467.356 (112 %)
2019	10.272.213 (121 %)	8.821.765 (99 %)	19.093.978 (110 %)

*ÄBD* ärztlicher Bereitschaftsdienst, *EBM* Einheitlicher Bewertungsmaßstab

**Table 3:** 

Jahr	Innerhalb der Sprechstundenzeit		Außerhalb der Sprechstundenzeit	
	Notaufnahme	ÄBD	Notaufnahme	ÄBD
2015	4.596.486 (100 %)	1.532.105 (100 %)	6.175.961 (100 %)	7.201.794 (100 %)
2016	4.584.256 (99,7 %)	1.252.117 (81,7 %)	6.486.669 (105,0 %)	7.566.116 (105,1 %)
2017	4.519.005 (98,3 %)	1.259.365 (82,2 %)	6.375.657 (103,2 %)	7.564.653 (105,0 %)
2018	4.474.813 (97,4 %)	1.269.260 (82,8 %)	6.306.854 (102,1 %)	7.834.907 (108,8 %)
2019	4.427.705 (96,3 %)	1.244.074 (81,2 %)	6.201.556 (100,4 %)	7.625.553 (105,9 %)

*ÄBD* ärztlicher Bereitschaftsdienst

Ab 2016 waren insbesondere die ambulanten Notfälle in den Krankenhäusern rückläufig

Als ambulante Notfälle wurden Behandlungsfälle mit mindestens einer Gebührenordnungsposition (GOP) aus Abschnitt 1.2 des Einheitlichen Bewertungsmaßstabs (EBM) gezählt. Im vierten Quartal 2014 wurde eine Differenzierung der GOP für innerhalb der Sprechstundenzeiten (GOP 01205, 01210 oder 01214) und außerhalb der Sprechstundenzeiten (GOP 01207, 01212, 01216 oder 01218) eingeführt. Sowohl ambulante als auch stationäre Notfälle umfassen eingewiesene und durch den Rettungsdienst eingelieferte Patienten und auch Selbsteinweisende (ausführliche Beschreibung der Datengrundlagen und der Methodik siehe [8]). Es fehlen Privatpatienten, berufsgenossenschaftliche (BG) Fälle und Verlegungen.

### Altersstruktur der Patienten

Berichte, dass ältere Patienten überproportional am ambulanten Fallzahlanstieg der Notaufnahmen beteiligt sind [10], werden durch die Analyse der ambulanten Abrechnungsdaten bestätigt. In Tab. 4 ist die Entwicklung der Anzahl und des Anteils an über 70-jährigen Notfallpatienten nach Versorgungsbereich von 2010 bis 2019 gezeigt. In den Notaufnahmen steigen sowohl die Anzahl als auch der Anteil hochbetagter Patienten unter den ambulanten Notfällen, während Anzahl und Anteil dieser Patientengruppe im ÄBD kontinuierlich abnehmen. Zwar ist der Anteil hochbetagter Patienten im ÄBD im gesamten Betrachtungszeitraum höher als in den Notaufnahmen, die Anzahl der über 70-jährigen Patienten in den Notaufnahmen übersteigt aber ab dem Jahr 2015 zunehmend die Anzahl der hochbetagten Patienten im ÄBD. Hiermit kann eine besondere Belastung der Notaufnahmen verbunden sein, die genauer analysiert werden muss. Dass das Problem erkannt ist, zeigen mehrere vom Innovationsfonds des G‑BA geförderte Projekte zur Verbesserung der Versorgung von Patienten mit Pflegestufe in Einrichtungen und in der Häuslichkeit (beispielsweise CoCare, Optimal@NRW, Treat@Home).

**Table 4:** 

Jahr	Notaufnahme		ÄBD	
	Anzahl Patienten > 70 Jahre	Anteil Patienten > 70 Jahre (%)	Anzahl Patienten > 70 Jahre	Anteil Patienten > 70 Jahre (%)
2010	865.540	11,9	1.266.790	18,6
2011	927.186	12,3	1.233.325	18,3
2012	980.915	12,8	1.243.628	18,5
2013	1.096.687	13,2	1.262.514	17,5
2014	1.147.572	13,4	1.163.357	17,1
2015	1.199.529	13,8	1.136.957	17,1
2016	1.223.893	13,7	1.080.928	16,1
2017	1.235.669	14,0	1.109.470	16,4
2018	1.261.568	14,4	1.123.981	16,1
2019	1.282.971	14,9	1.076.349	15,8

*ÄBD* ärztlicher Bereitschaftsdienst

## Diskussion

Zunächst stellt sich die Frage nach dem Erfolg der bisherigen Maßnahmen. Nach Angaben der Kassenärztlichen Bundesvereinigung wurden zwischen 2015 und 2019 rund 620 Bereitschaftspraxen in einer Notaufnahme oder in unmittelbarer Nähe dazu eingerichtet. Damit existiert rechnerisch an jedem zweiten der rund 1200 Krankenhäuser mit einer Notfallstufe gemäß § 136c Abs. 4 SGB V eine Bereitschaftspraxis. Eine Ausweitung dieses Angebots auf alle Krankenhäuser mit Notfallstufe dürfte wenig effizient sein, da die Mehrheit der Notaufnahmen hierfür keine ausreichenden Fallzahlen aufweist. Nur rund ein Drittel aller Notaufnahmen, die ambulante Fälle gegenüber KVen abrechnen, generiert mehr als einen ambulanten Abrechnungsfall pro Stunde [11]. Die Effekte der bisher eingerichteten Bereitschaftspraxen lassen sich anhand der Abrechnungsdaten nicht zweifelsfrei nachweisen. Erste Hinweise legen jedoch nahe, dass rückläufige Fallzahlen ambulanter Notfälle insbesondere an Krankenhäusern mit Bereitschaftspraxen beobachtet werden können [11]. Dafür spricht auch, dass gemäß der dokumentierten Abrechnung eine überwiegend klare und über die Jahre stabile Arbeitsteilung zwischen Bereitschaftsdienst und Notaufnahmen besteht. Während in den Notaufnahmen verletzungsbedingte Diagnosen dominieren, sind es im ÄBD Infektionskrankheiten sowie typisch geriatrische Erkrankungen [11, 12]. Ausstattung und Besetzung der Bereitschaftspraxen sowie die Kooperation zwischen Notaufnahmen und Bereitschaftspraxen sind jedoch heterogen. Zudem muss von einer Sogwirkung eines entsprechenden Angebots ausgegangen werden. Diese Komplexität besteht auch international und erschwert die Bewertung des Effekts [13].

Rechnerisch existiert an jedem zweiten Krankenhaus mit Notfallstufe eine Bereitschaftspraxis

Die Abrechnungsdaten zeigen, dass Notfallbehandlungen in Notaufnahmen vor allem während der Sprechstundenzeiten zu beobachten sind (Tab. 3). Eine Dokumentation des Behandlungszeitpunkts bei ambulanten Notfällen wurde 2015 eingeführt. Die Einführung einer finanziellen Förderung der Behandlung von Akutfällen in der vertragsärztlichen Versorgung und einer 24 h täglich und 7 Tage in der Woche erreichbaren Servicestelle der KVen zur Vermittlung von Versorgungsangeboten für Akutfälle begann 2020. Das Zentralinstitut für die kassenärztliche Versorgung in der Bundesrepublik Deutschland (Zi) veröffentlicht seit März 2022 Daten zu Anzahl und Anlässen der Ersteinschätzung [14]. Demnach finden derzeit monatlich rund 130.000 telefonische Ersteinschätzungen statt. Der Effekt auf die Direktinanspruchnahme der Notaufnahmen ist – auch infolge der Überlagerung durch die Pandemie – bisher nicht statistisch nachgewiesen worden. Die Mehrheit der Anrufer nutzt bislang das Angebot der KVen zu Bereitschaftsdienstzeiten, vor allem dann, wenn auch Bereitschaftspraxen besetzt sind.

Die in Diagnosen erkennbare Arbeitsteilung mit den Bereitschaftspraxen signalisiert, dass diese als weitgehend etablierte Praxis angesehen werden kann. Punktuelle Verbesserungen bei der technischen Ausstattung und der Besetzung werden diskutiert. Im Raum stehen insbesondere Labor, bildgebende Verfahren/Sonographie und Wundversorgung. Weitere Anforderungen können aus der zunehmenden Zahl von Akutfällen bei Hochbetagten abgeleitet werden, beispielsweise in Bezug auf den Wechsel von Blasenkathetern.

Die Beratungen des G‑BA zu § 120 Abs. 3b SGB V problematisieren die Patientensteuerung während der Praxisöffnungszeiten. Akutpatienten müssen dann zur Behandlung in eine Praxis außerhalb des Krankenhausgeländes geleitet werden. Kritisch wird diskutiert, welches Verfahren eine sichere Ersteinschätzung als Grundlage der Weiterleitung bietet, da die zur zeitlichen Priorisierung in Notaufnahmen eingesetzten Verfahren, beispielsweise das Manchester Triage System (MTS), dafür allein nicht ausreichend sein dürften [15]. Denkbar wäre eine Kombination mit einem Telefontriageverfahren [16, 17]. Tatsächlich findet sich in der Literatur bislang kein etabliertes Verfahren zur Unterstützung von Steuerungsentscheidungen hinsichtlich der Weiterleitung von einer Notaufnahme in eine räumlich entfernte Einrichtung zur ambulanten Versorgung. In Deutschland werden derzeit zwei Verfahren in klinischen Studien evaluiert [18–20]. Liegen die Ergebnisse vor, wird auch erneut zu beraten sein, ob vor jeder Weiterleitung zwingend auch eine ärztliche Prüfung erforderlich ist. Zudem benötigen die Notaufnahmen technische und personelle Unterstützung, um die Terminanmeldung in Praxen schnell und effizient erledigen zu können.

In Notaufnahmen ist Überlastung (Crowding) mit erhöhten Risiken für vermeidbare Komplikationen und Mortalität bei der Behandlung der Notfälle verbunden [21]. Aufgrund der Komplexität des Crowdings mag der Beitrag durch Entlastung um Akutfälle umstritten sein [22, 23]. Geeignete Lösungen müssen insbesondere für ältere, multimorbide Patienten gefunden werden [21]. Im deutschen Kontext muss jedoch geklärt werden, wann Versicherte berechtigterweise eine Behandlung in der Notaufnahme in Anspruch nehmen. Nach geltender Rechtsprechung sind die Notaufnahmen im Rahmen der ambulanten Behandlung stets auf das Leistungsspektrum des Bereitschaftsdiensts beschränkt [24]. Diese Vorgabe kontrastiert oft mit dem diagnostischen Vorgehen der Notfallmedizin, das davon ausgeht, dass Patienten, die eine Notaufnahme aufsuchen, stets besondere Gesundheitsrisiken aufweisen. Fraglich ist, ob die Einrichtung von INZ geeignet sein kann, diesen Widerspruch aufzulösen. Von den 10,6 Mio. ambulanten Notfallbehandlungen der Notaufnahmen im Jahr 2019 entfielen 4,4 Mio. auf Praxisöffnungszeiten. Aus Sicht der Krankenhäuser ist das eine relevante Größenordnung. Allerdings wurden nach Schätzungen des Zi zugleich rund 200 Mio. Akutfälle in den Praxen behandelt [12]. Versicherte wenden sich im Akutfall zu Praxisöffnungszeiten somit ganz überwiegend an niedergelassene Praxen. Dies verdeutlicht aber das Verlagerungspotenzial, das bei einer Einführung von INZ Entlastungseffekte für diese Standorte zunichtemachen könnte. Dies dürfte insbesondere dann der Fall sein, wenn INZ künftig nicht mehr an Restriktionen des Bereitschaftsdiensts gebunden sein sollten und auch eine personelle und sachliche Ausstattung erhalten würden, die eine fachübergreifende Regelversorgung nach dem Prinzip „alles aus einer Hand zu jeder Tages- und Nachtzeit“ ermöglicht.

Ein solcher Ansatz wäre angesichts des Fachkräftemangels nur durch erhebliche Umstrukturierung der ambulanten und stationären Regelversorgung realisierbar. Wie sehr ein solches Konzept aber vom Zahlengerüst der Versorgungsrealität entfernt ist, wird anhand erster Ergebnisse einer Machbarkeitsstudie zur Patientensteuerung am Klinikum Rosenheim deutlich: Im Beobachtungszeitraum waren zwei Drittel der eingeschlossenen Patienten selbsteinweisend. Davon konnte rund die Hälfte vertragsärztlich behandelt werden. Diese Behandlung erfolgte zu mehr als 95 % durch die Bereitschaftspraxis, weil selbsteinweisende Patienten vor allem zu diesen Zeiten die Notaufnahme aufsuchten. Für weniger als 5 % der selbsteinweisenden Patienten kam eine Weiterleitung in eine Vertragsarztpraxis abseits des Klinikgeländes infrage. Konkret betraf dies in einer Notaufnahme, die mit rund 45.000 Patienten jährlich mehr als das Doppelte der mittleren Patientenzahl aufweist, im Zeitraum eines Monats 24 Patienten. Diese Zahl kann nach Einschätzung der Beteiligten der Machbarkeitsstudie gesteigert werden, wenn ressourcenbedingte Zuordnungen an die Notaufnahme überprüft und eingelieferte Patienten einbezogen werden. Insgesamt dürfte auch dieses Potenzial vertragsärztlicher Entlastungangebote insgesamt bei überschaubaren Zahlen bleiben: Im Beobachtungszeitraum der Machbarkeitsstudie wurden auch zu den Tageszeiten mit hoher Belastung im Schnitt nicht mehr als 9 Patienten pro Stunde erreicht, wovon im Mittel knapp 2 Patienten der vertragsärztlichen Versorgung zugeordnet wurden. Man muss sich dieser Größenordnung bewusst sein, wenn man über die Einführung von INZ als einem zusätzlichen Versorgungsangebot reflektiert. Effiziente INZ-Lösungen dürften daher nur an sehr wenigen Standorten existieren, in der Breite müssen sie in einer Patientensteuerung mit klaren Zuständigkeiten bestehen, die durch geeignete Ersteinschätzungsverfahren unterstützt wird [25].

## Fazit für die Praxis

- Noch wird an der Umsetzung bereits erfolgter gesetzlicher Interventionen zur Entlastung der Notfallversorgung von vertragsärztlich behandelbaren Patienten gearbeitet. Diese fokussieren auf praktikable Lösungen zu einer Patientensteuerung, die auf strukturierten Ersteinschätzungsverfahren aufsetzt.
- Der aktuelle Koalitionsvertrag formuliert weitergehenden Handlungsbedarf im Bereich der Notfallversorgung. Anhand der Auswertung von Daten zur Notfallversorgung wird im vorliegenden Beitrag empfohlen, vor weiteren Reformen bisher erarbeitete Lösungsansätze zu testen und zu evaluieren. Das jüngst mit Datum vom 12.07.2022 bekannt gewordene Schreiben des Bundesgesundheitsministeriums an den G‑BA mit der Ankündigung, dass die Frist zur Entscheidung über die Richtlinie Ersteinschätzung um ein Jahr zu verlängert werden soll, setzt das richtige Signal dafür.
